# An Analysis of Polymer Gear Wear in a Spur Gear Train Made Using FDM and FFF Methods Based on Tooth Surface Topography Assessment

**DOI:** 10.3390/polym13101649

**Published:** 2021-05-19

**Authors:** Jadwiga Pisula, Grzegorz Budzik, Paweł Turek, Mariusz Cieplak

**Affiliations:** 1 Faculty of Mechanical Engineering and Aeronautics, Rzeszów University of Technology, 35-959 Rzeszów, Poland; gbudzik@prz.edu.pl (G.B.); pturek@prz.edu.pl (P.T.); 2 CC Metal, 38-100 Strzyżów, Poland; m.cieplak@interia.eu

**Keywords:** spur gears, FDM, ABS, ULTEM, PEEK, wear, topography of gear tooth

## Abstract

This article focuses on wear tests of spur gears made with the use of additive manufacturing techniques from thermoplastic materials. The following additive manufacturing techniques were employed in this study: Melted and Extruded Modelling (FDM) and Fused Filament Fabrication (FFF). The study analysed gears made from ABS M-30 (Acrylonitrile Butadiene Styrene), ULTEM 9085 (PEI Polyetherimide) and PEEK (Polyetheretherketone), and the selection of these materials reflects their hierarchy in terms of economical application and strength parameters. A test rig designed by the authors was used to determine the fatigue life of polymer gears. Gear trains were tested under load in order to measure wear in polymer gears manufactured using FDM and FFF techniques. In order to understand the mechanism behind gear wear, further tests were performed on a P40 coordinate measuring machine (CMM) and a TalyScan 150 scanning instrument. The results of the gear tests made under load allow us to conclude that PEEK is resistant to wear and gear train operating temperature. Its initial topography undergoes slight changes in comparison to ABS M-30 and Ultem 9085. The biggest wear was reported for gears made from Ultem 9085. The hardness of the material decreased due to the loaded gear train’s operating temperature.

## 1. Introduction

Additive manufacturing is increasingly used in the electromechanical industry and reconstructive medicine [1]. Its popularity is a result of constantly improved additive techniques and materials as well as the possibility of manufacturing non-standard products of complex geometries, sometimes unattainable by conventional methods, but primarily thanks to lower production costs due to shorter manufacturing lead times without the need to use tools or instruments. Additive manufacturing has its drawbacks, which include mainly limitations with regard to low-volume production or small-sized parts, as well as the absence of data concerning final properties of the product (strength, thermal stability, etc.). The authors of the publication suggest that the design of gears made with AM methods should use data directly from the tested gears, not from simple standard specimens [2].

Gears are machine parts commonly used in drive trains. Since studies on gears and gear trains are most frequently performed with reference to gears manufactured using conventional methods from metal alloys, they have been widely discussed in the literature. Most recent studies focus mainly on gear trains of advanced structures and the optimisation of their designs in terms of strength by means of kinematic models [3,4,5], dynamic models [6,7,8] and diagnostics [9,10,11,12,13,14]. There are also publications providing an overview of gear design tools with guidelines and practical examples of gear design [15]. Polymer gear and polymer gear train studies have been published, but the number of these is much smaller than in the case of conventional technologies and materials. The authors of such publications present results mostly concerning spur gears. The studies mainly discuss various aspects of gear train modelling, taking into consideration polymer gear meshing stiffness with the use of numerical methods (the finite element method (MES)) and analytical techniques [16,17]. Publications contain guidance on the design of polymer gears intended to improve gear train performance and durability [18]. Authors also concentrate on testing the strength of polymer spur gears with consideration of their tribological properties when operating under load without lubrication [18,19,20,21,22,23]. Few publications discuss the operation of lubricated polymer gears [24]. For the purpose of the studies, gears are manufactured by means of selected techniques. Most frequently, they include polymer and composite polymer injection-moulded or machined gears [18,19,20,21,22,23,24]. The literature does not contain information on studies analysing wear resistance of additive manufactured gears, except for the assessment of their geometrical accuracy [25,26,27,28,29]. Many authors present results of the analyses obtained from strength tests on samples of materials used in additive manufacturing. Their studies focus on the selection of suitable technological parameters of the additive manufacturing process and the optimisation of source model geometry in order to provide maximum strength and required geometrical accuracy of the product [30,31,32,33,34,35].

Coordinate metrology plays an important role in the manufacturing process. Its purpose is to verify whether the item has been manufactured in accordance with specifications, e.g., with reference to geometrical accuracy [25,26,27,28,29,30,31,32,33,34,35,36,37,38] and the condition of the geometrical surface structure (mostly surface roughness) [39,40,41]. A broad spectrum of studies evaluating the condition of the surface layers of parts manufactured using additive techniques have been recently published [42,43,44]. The geometrical structure of the surface of models made using additive manufacturing techniques is the effect of multiple factors [44,45,46,47]. The most significant ones include the printing method applied [46,48,49], the orientation of the part in the printer’s build space [44,47], as well as post-processing [45,46]. Prior to surface layer condition assessment for models made by additive manufacturing techniques, it is important to develop a methodology allowing for the choice of a suitable measuring method and analysis of results [43]. There are various methods of measuring quantities describing the shape of the surface layer [50,51,52]. These include profile (horizontal and vertical scanning) and surface methods. Profile methods are used for analysing the geometrical structure of the surface of additive manufactured parts. Horizontal scanning systems include contact systems with or without a skid [41,42,43,44]. There are a number of non-contact methods of verifying the condition of the surface layer using vertical scanning. The most frequently used ones include: confocal microscopy [53,54,55], structured light projection [56,57], focal differentiation microscopy [58,59,60] or coherence scanning interferometry [61]. Although increasingly modern optical methods of surface geometrical structure measurements are widely available, contact methods are still superior in terms of measurement repeatability [41,50].

This article presents the results of experimental wear resistance tests performed on spur gears made by means of additive manufacturing with the use of material extrusion. The additive manufacturing techniques employed in this study are Melted and Extruded Modelling (FDM) and Fused Filament Fabrication (FFF). Gear teeth materials were appropriately selected for their application and the additive manufacturing technique of choice. These included ABS M-30 (Acrylonitrile Butadiene Styrene), ULTEM 9085 (PEI Polyetherimide) and PEEK (Polyetheretherketone). The subject of the present study was chosen due to the absence of publications on wear in gears made using additive manufacturing techniques.

## 2. Materials and Methods

### 2.1. Research Object

A pair of spur gears were designed in order to run fatigue tests on cylindrical gear trains; the parameters are listed in Table 1. A mathematical model of teeth obtained in the envelope machining process using rack tooth profile. A tool defined according to ISO 53:1998 [62] was applied to make 3D CAD (Computer-aided design) models. Gear teeth were without addendum modification. An assumed value of the circumferential backlash of 0.2 mm was taken into account on the rack tool profile used to form the gear gap. All the allowance was provided on the mating gear. The tooth space profiles for both gears were generated with a high accuracy (about 300 points (knots) were used for spline interpolation). Based on the profiles, 3D CAD gear models were designed and subsequently converted into the STL (the STereoLithography (STL) file) format supported by additive manufacturing machines.

The gear pair was placed in a two-part gear train housing, which had been manufactured by additive techniques from ABS. The gear train featured spline shafts with external diameters of, respectively, 16 and 25 mm (KW13 × 16, KW21 × 25), supported in the housing by ball bearings.

### 2.2. Materials

The gears were made from the following polymer materials: ABS, PEI and PEEK. The materials are described as plastomers, more precisely as thermoplastics, and become pliable above a certain temperature referred to as the processing temperature. These properties enable the material to be worked in a plastic state, moulded into the expected shape and solidified by cooling. The structure of thermoplastics can be amorphous, i.e., composed of randomly distributed polymer chains (ABS, PEI), or semi-crystalline, if it contains partly aligned particles (PEEK). The choice of materials for test gears was made on the basis of their intended application. ABS is a polymer for consumer products; it is inexpensive and widely available. In contrast, PEI and PEEK belong to a group of technically advanced polymers. Among the materials mentioned above, PEEK has optimum properties as it offers good resistance to abrasive wear, retains dimensional stability in high temperatures and resistance to most chemical reagents as well as penetrating radiation such as gamma rays. PEI and ABS feature inferior resistance to abrasive wear compared to PEEK, while ABS clearly offers the lowest abrasive wear resistance. Tests were performed on gears made from the following materials: ABS M-30 (Stratasys Inc., Eden Prairie, MN, USA), ULTEM 9085 (Stratasys GmbH, Frankfurt, Germany) and PEEK (360–400) (3D4Makers BV, Haarlem, The Netherlands). Table 2 presents selected properties of materials used.

### 2.3. Additive Manufacturing

The use of a specific materials is related to the selection of additive manufacturing technique. For this reason, the following additive manufacturing techniques were used to make gear models from thermoplastic materials: FDM (Melted and Extruded Modelling) and FFF (Fused Filament Fabrication). Available printers also determine or radically limit the choice of the material for printed models. The article presents gears made from three printers. Test model print details are supplied in Table 3.

The FDM technique was applied to make gear pairs from materials ABS M-30 and ULTEM 9085. The FDM technique involves building parts by applying consecutive layers of a semi-liquid thermoplastic. The process takes place in a suitably high temperature to minimise deformations due to linear shrinkage of the material. “FDM” is a name patented by Stratasys. Manufacturers of printers utilizing this technique use the name FFF (Fused Filament Fabrication). The FFF technique was applied to make gear pairs from PEEK.

In this study, Stratasys F170 (Stratasys Inc., Eden Prairie, MN, USA) printer was one of additive manufacturing machines using the FDM technique [67]. The models were built from ABS M30 in the form of a 1.75 mm thick filament. The F170 printer is characterised by a completely enclosed build space. The machine is a dual extruder printer, which allows support structures to be printed from a material soluble in a specific solution. The printer’s build space is sized at 254 × 254 × 254 mm. It has the capability to apply material in four height variants: 0.330, 0.254, 0.178, 0.127 mm. During the process, a stable temperature of 80°C was maintained in the test chamber. Gears were made with a layer thickness of 0.254 mm, with a solid fill material and two profiles.

A Fortus 450mc printer using FDM technology was applied to manufacture gears from a thermoplastic composite polymer named ULTEM 9085. The Fortus 450mc printer (Stratasys Inc., Eden Prairie, MN, USA) is an industrial machine sized at 406 × 355 × 406 mm [68]. The printer features two separate heads, one for the base material and one for support material (dual extrusion design); the temperature inside the build chamber is 110 °C. Possible layer thicknesses for ULTEM 9085 are 0.330 or 0.254 mm.

An Industry 340 3DGence printer [69] (3DGence, Przyszowice, Poland) utilizing FFF technology was used to fabricate models from the PEEK material for analysis. The printer’s build chamber is sized at 260 × 300 × 340 mm. The printer features a single head with a dual extrusion system; nozzle diameter—0.4 mm, filament diameter—1.75 mm, head temperature—up to 500 °C, build table temperature—up to 160 °C, build chamber temperature—up to 85 °C, filament chamber temperature—up to 70 °C, minimum layer thickness—0.04 mm. In the analysed case for PEEK, the lowest thickness value applied was 0.15 mm.

**Figure 1:**
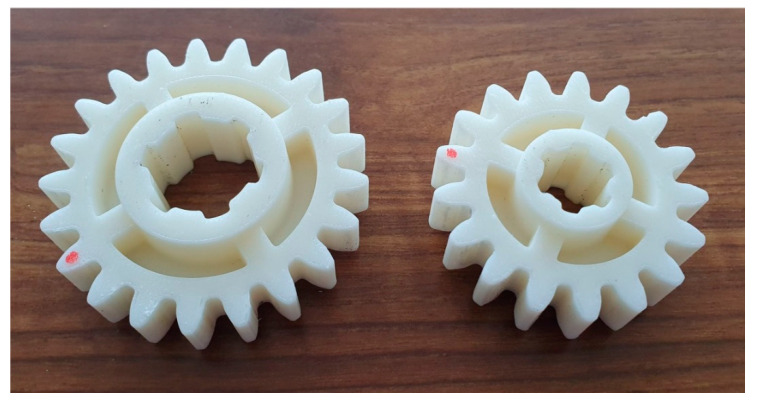
Gears manufactured using the FDM technique from ABS M-30.

**Figure 2:**
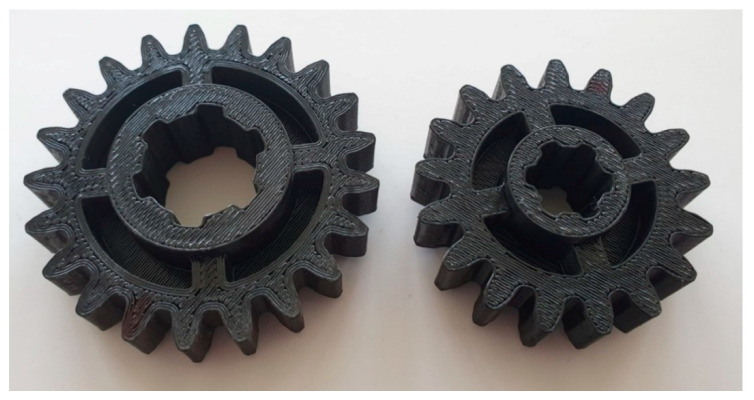
Gears manufactured using the FDM technique from ULTEM 9085.

**Figure 3:**
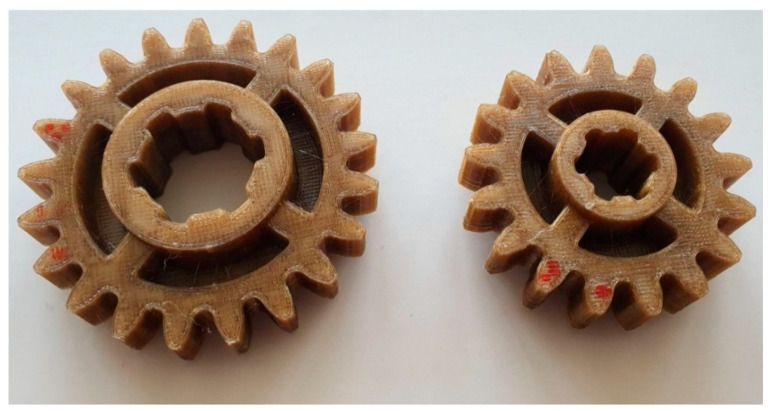
Gears manufactured using the FFF technique from PEEK.

Each pair of gears from a specific material was made in a set of 10 pcs. Figure 1, Figure 2 and Figure 3 present selected gear pairs made, respectively from ABS M-30, Ultem 9085 and PEEK.

### 2.4. Gear Fatigue Test Rig

**Figure 4:**
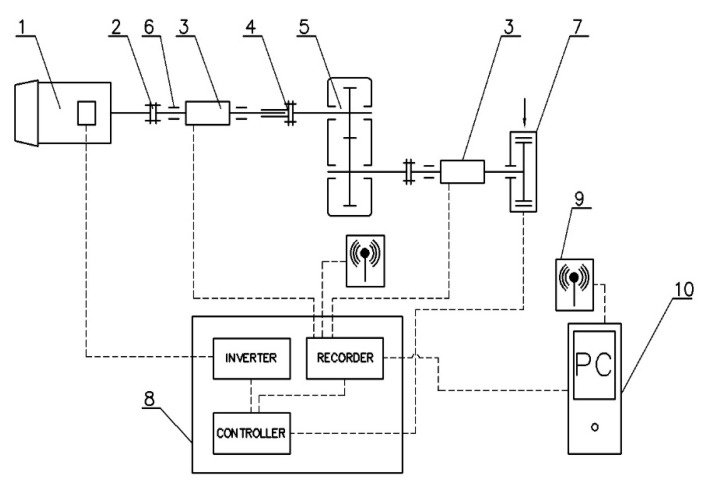
An outline of the rig for carrying out fatigue tests on cylindrical gears. Key: 1—motor, 2—clutch, 3—torque meter, 4—disengaging clutch, 5—analysed gear train, 6—bearing support, 7—powder brake, 8—control box, 9—WiFi connectivity, 10—computer.

Figure 4 presents an outline of the rig for carrying out fatigue tests on cylindrical gears made from polymer materials.

**Figure 5:**
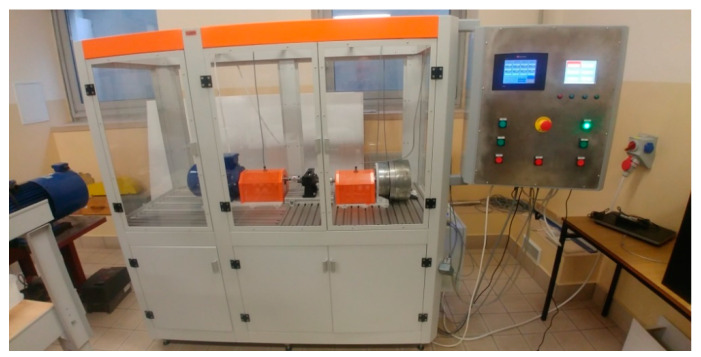
Fatigue test rig for polymer gears.

The test rig was of an open design. The rig was powered by a three-phase 2.2kW motor (type MS 112M-6, the nominal speed 955 rpm, the nominal torque 22 Nm), the speed of which ranges from 200 to 2000 rpm, with variable control by a three-phase power inverter. The rig was equipped with two torque meters, connected directly to the RMC monitor, enabling real-time readouts of torque and speed values (sampling frequency—10 Hz). On the output, a powder brake with variable load adjustment (up to 70 Nm) was installed. Data were acquired with the RMC-M software, offering a preview of the measurement results and a printout in the form of a report (Figure 5). The first stage of the rig can be loaded up to 20 Nm (due to the motor used), and the maximum load at the output is 50 Nm. Torque meter resolution—0.1%.

The test rig was also fitted with a Micro-Epsilon SN8023284 pyrometer, connected to the RMC monitor.

### 2.5. Rig Tests on the Gear

The main failure observed during the operation of gears made of polymer materials are: tooth cracks or breakage, tooth deformation, removal of tooth material (change in active tooth profile and tooth thickness), and surface fatigue. Wear detection is the most common method of detecting failure of polymer gears.

**Figure 6:**
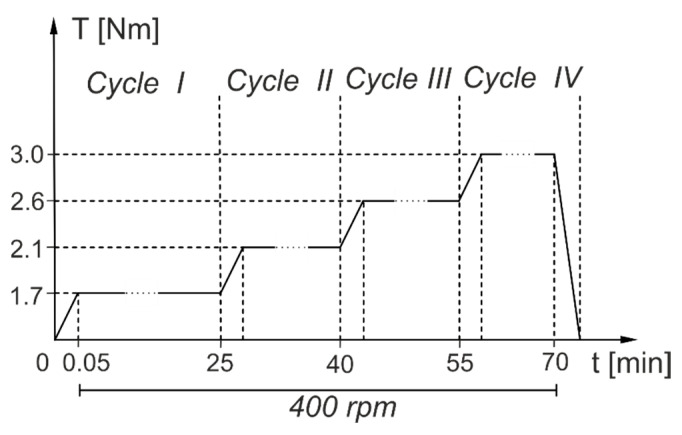
A chart presenting a complete load cycle applied at the test rig.

Tests were performed in the same conditions, with the same input parameters for all gear pairs made of thermoplastics shown in Figure 1, Figure 2 and Figure 3. The test regime was based on experiments which allowed us to determine the value of the destructive load for an ABS gear. Figure 6 presents the load chart for a complete work cycle to which gear pairs were subjected. The test rig controller was programmed for 4 load cycles fixed at 400 rpm.

### 2.6. Tooth Surface Geometrical Accuracy Measurements

**Figure 7:**
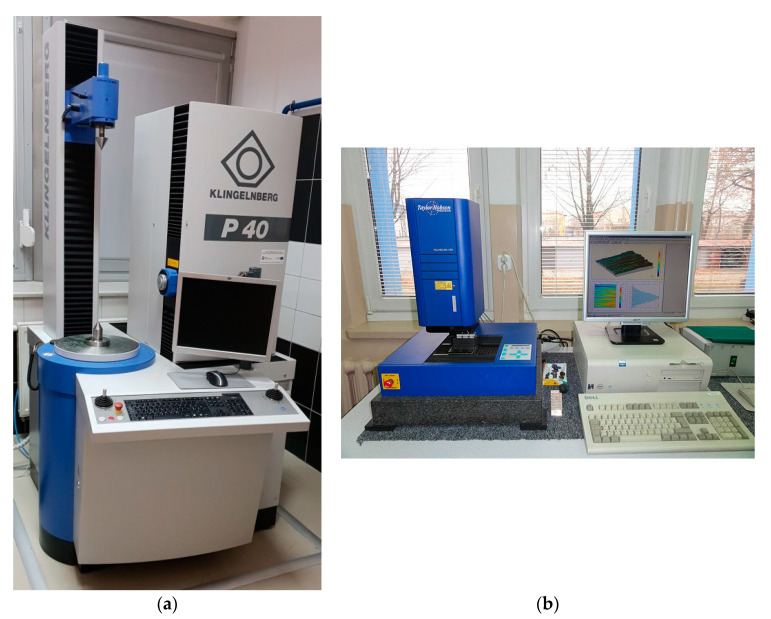
Tooth surface topography assessment instruments: (**a**) P40 CMM; (**b**) TalyScan 150.

Gear tooth flank geometrical accuracy measurements were performed in accordance with measurement methodology on a Klingelnberg P40 (Hückeswagen, Germany) coordinate measuring machine as well as TalyScan 150 scanning instrument by Taylor Hobson (Leicester, UK) (Figure 7).

#### 2.6.1. Measurements on the P40 CMM

Gear model measurements were taken using Klingenberg’s P40 CMM used as a test centre for gears [70]. The measurements were performed in accordance with spur gear measurement methodology on a coordinate measuring machine with the use of specialist software, GINA. The measurements covered spur gear teeth and were taken after the manufacturing process (printing and post-processing), as well as after tests performed on a fatigue test rig.

Measurements were performed in laboratory conditions at the temperature from 18 °C to 20 °C and constant humidity. A rod with two ruby tips with diameters of 1.5 mm each was used in the measurements (Table 4).

During measurement, two suitably selected reference bases (cylindrical and face surface) for both gears were used. The gears were placed in a three-jaw chuck in the centre point of the rotary table of the P40 coordinate measuring machine. Gear measurements were assessed in compliance with DIN 3962 [71], and measurement results were presented in the form of measurement sheets.

#### 2.6.2. Tooth Flank Surface Geometry Measurements

Tooth flank surface geometry measurements were performed by means of Taylor Hobson’s TalyScan 150 scanning instrument (Leicester, UK) with a 2 μm stylus tip radius. In the surface roughness assessment process, the sampling step on X and Y axes was set at minimum value equal to 5 μm. Each measured area was sized at 4 × 2 mm. Lowest available measurement speed of 500 µm/s was applied during the measurement. As the samples were manufactured by means of an additive technique, the greatest changes in the height of rough surfaces were observed to be perpendicular to the direction of layer application. Accordingly, the tests were carried out along the perpendicular direction. The data collected were analysed in the MountainsMap software. The first stage of the process of determining surface roughness parameters involved the correction of shape errors using a 3 degree polynomial. Next, in order to isolate long-wave components, a profile filter λc = 0.8 mm was applied. The length of the roughness sample cut-off was determined on the basis of the procedure for periodic roughness profiles specified in ISO 4288 [72]. The filtering process led to the limitation of the test area to 3.2 × 1.2 mm, in reference to which selected stereometric parameters were determined according to ISO 25178-2 [73]:

- Arithmetical mean of absolute values of ordinates within the defined area (A)—*S_a_*:
  (1) $$S_{a}=\frac{1}{A}\overset{}{\underset{A}{\iint }}\left|z\left(x,y\right)\right|dxdy$$
- Root-mean-square of absolute values of ordinates within the defined area (A)—*S_q_*:
  (2) $$S_{q}=\sqrt{\frac{1}{A}\overset{}{\underset{A}{\iint }}z^{2}\left(x,y\right)dxdy}$$
- Greatest value of the height of the apex within the defined area—*S_p_*;
- Greatest value of the depth of the concavity within the defined area—*S_v_*;
- The sum of the greatest value of the height of the apex and the greatest value of the depth of the concavity within the defined area—*S_z_*:
  (3) $$S_{z}=S_{v}+S_{p}$$
- Quotient of the mean value of cube ordinates and cube *S_q_* within the defined area (A)—*S_sk_*:
  (4) $$S_{sk}=\frac{1}{S_{q}^{3}}\left[\frac{1}{A}\overset{}{\underset{A}{\iint }}z^{3}\left(x,y\right)dxdy\right]$$
- Quotient of the mean value of fourth power ordinates and fourth power *S_q_* within the defined area (A)—*S_ku_*:
  (5) $$S_{ku}=\frac{1}{S_{q}^{4}}\left[\frac{1}{A}\overset{}{\underset{A}{\iint }}z^{4}\left(x,y\right)dxdy\right]$$

## 3. Results and Discussion

**Figure 8:**
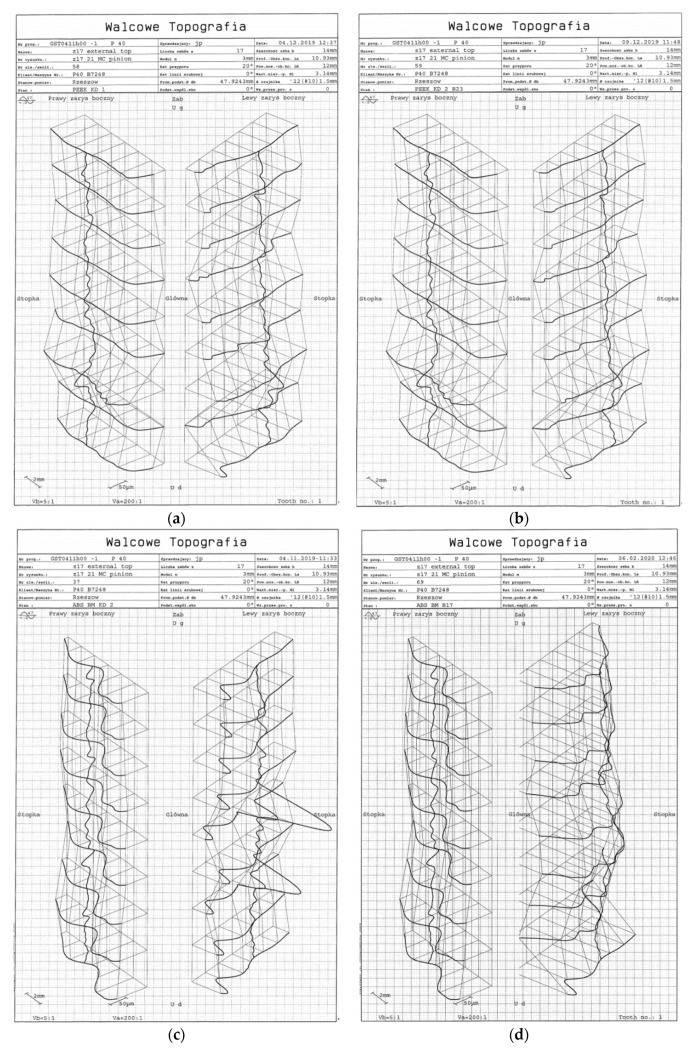

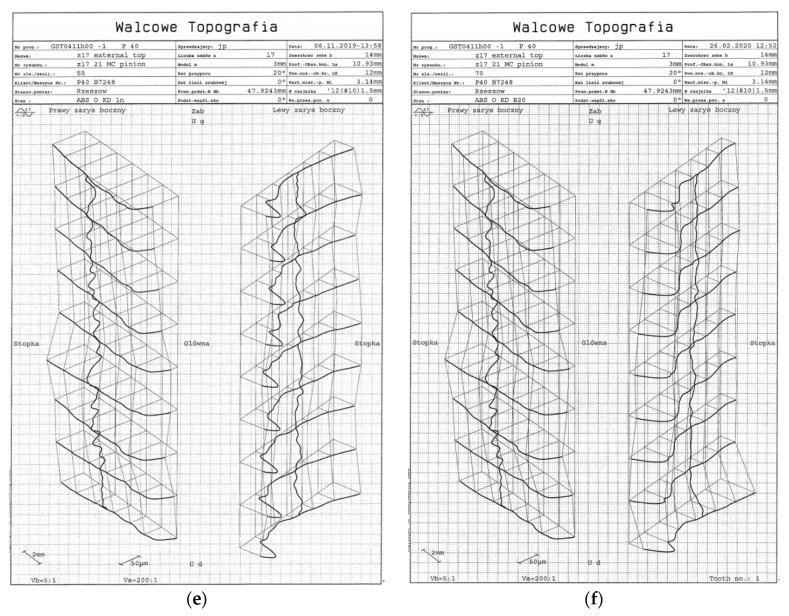
Results of topography measurement for pinion z17 before and after tests for selected representative samples (**a**) PEEK pre-test; (**b**) PEEK post-test; (**c**) ULTEM 9085 pre-test; (**d**) ULTEM 9085 post-test; (**e**) ABS M-30 pre-test; (**f**) ABS M-30 post-test.

This study assessed the topography of tooth flank surfaces of each test gear, both after printing and after the required load cycle on the fatigue test rig. Tooth flank topography was defined by tooth profile and tooth trace measurement was distributed evenly across the width of the tooth and its height from the root cylinder to the tip cylinder. In the analysed case, tooth topography was determined on the basis of the measurements of nine profiles distributed evenly over the profile assessment interval Lα (10.93 mm), defined according to the standard, and seven tooth traces located within the tooth trace assessment interval Lβ (12 mm), defined according to the standard (Figure 8). The tooth topography chart demonstrates deviation distribution measured on the tooth profile and tooth trace from the lowest value, treated as a reference, to the highest value obtained. This article presents topography for the selected tooth, respectively, for all pinions (z = 17) before and after the load cycle (Figure 8).

After printing, pinion (z17) tooth flank topography was different on the right-hand side and the left-hand side of the tooth. ABS M-30 pinions have diverse topographies; on the right-hand tooth flank, the deviations were equal across the entire width of the tooth, and they decreased across its length near the tip (Figure 8e). The highest deviation values appeared on the left-hand flank at the tip; they dropped abruptly and formed a valley, to gradually increase at the foot. Ultem 9085 gears display an uneven distribution of deviations across the height of the tooth (Figure 8c). The greatest deviations were observed for the tooth tip. They formed a concavity across the tooth width on the right-hand flank, at the middle height of the tooth. On the left-hand flank near the tip, the deviations decreased but returned to higher values later on. The right-hand flank had the same deviations across the entire width of the tooth, while the left-hand one was uneven and contained peaks. PEEK gears feature relatively identical distributions across the length of the tooth on both flanks, with the right-hand side displaying identical deviation values except for the region of the tip, where the deviations were the lowest; on the left-hand side, starting from mid-height of the tooth, the values drop slightly towards the tooth tip (Figure 8a). Topography charts of printed gear wheels (z21), not featured in this article, preserve the characteristics of the pinions.

Gear accuracy was measured after the test in which the gears were subjected to the designed load cycle. It was reported that the gears, both gear wheels and pinions, were outside accuracy class 12 in all cases. This was caused by deterioration of parameters describing profile geometry, tooth trace, pitch and thickness. The smallest changes were captured for gears made using the FFF technique from PEEK. Tooth flank topography of surfaces obtained after the designed load cycle at the fatigue load test rig was assessed for changes which had been caused by gear meshing under load. Gear meshing in the gear train was unilateral and unidirectional (in accordance with the direction of the rotary movement transferred from the motor to the gear train by the drive shaft). Consequently, topography charts indicate that only one tooth flank changed its geometry. According to the results for pinion topography (z = 17), this was the right-hand side of the tooth flank. Deviations in the right-hand side topography of the tip of the ABS M-30 gear tooth following tests were significantly neutralised in comparison with the remaining surface of the tooth flank with an even distribution of deviations (Figure 8f). In the case of Ultem 9085, peaks were removed from the topography of the meshing side, yet at the same time the tooth tip was worn, along with the area at the base of the tooth (Figure 8d). As regards FDM gears, the deviations on one end across the width of the tooth were clearly not removed. This is caused by different widths of the teeth of meshing gears and a non-symmetrical placement of their toothed rings relative to each other. As for FFF gears, the topography of the meshing side was slightly smoothed out (Figure 8b).

Gear tooth flank surface topography measurements enabled tooth geometry assessment across the entire width of the toothed ring. In addition, it allowed us to compare the topography of teeth obtained after the gear teeth manufacturing process with the topography of the same teeth after the load cycle described above. An analysis of topography charts after the manufacturing process (printing, stabilisation and post-processing) made it possible to define geometric defects on tooth flank surfaces resulting from the printing process. For the FDM technique and ABS M-30 and Ultem 9085 materials, the left-hand flank of a pinion tooth contained a clearly visible bulge at the tip. PEEK gears made using the FFF technique ensured a more stable printing process, as confirmed by the relatively even distribution of deviations in both directions, across the width and the height of the tooth.

**Figure 9:**
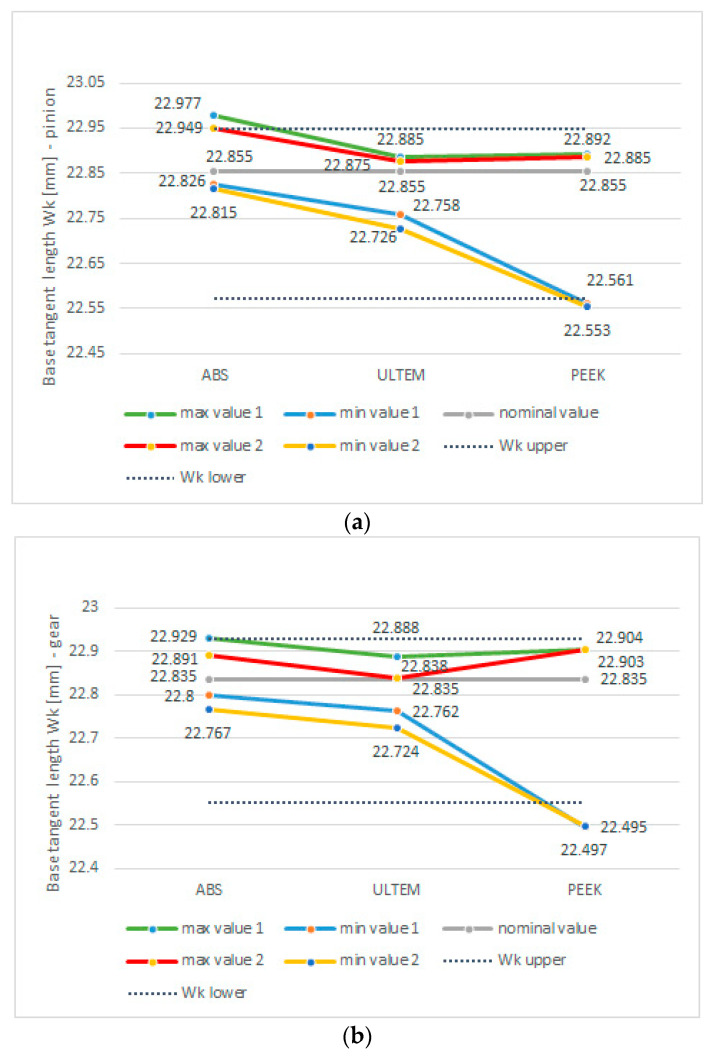
Base tangent length over k teeth for: (**a**) pinion; (**b**) gear wheel, 1—after printing, 2—after test rig.

In order to achieve a better understanding of the phenomenon of tooth flank wear, it is necessary to refer to tooth thickness, which has a significant effect on backlash, in particular due to the heat expansion of polymer materials, responsible for dimensional instability. The designed backlash for the test gear allowed for heat expansion as well as the accuracy of the additive manufacturing process. Figure 9 presents tooth thickness measurement for gears after printing and tests using Wk measurement length. Note that the tooth backlash has been considered in the driven gear model. Thus, the value of Wk measurement length for the driven gear was reduced by the value of the nominal normal tooth clearance (Wk = 22.835 mm). The greatest thickness was reported for gears made from ABS M-30. Although thickness in excess of the nominal value with allowance was obtained, there was a backlash, preventing the gears from interfering. The value of the backlash was increased by the positive deviation of the centre distance by the circumferential backlash value of 0.036 mm. The actual centre distance was 57.05 mm and was caused by inaccuracies in the manufacturing of the frame by FDM additive technology. The largest thickness fluctuations were observed for gears made from PEEK, which suggests dimensional instability of the manufacturing process. Tooth thickness fluctuations for ABS M-30 and Ultem 9085 were two times smaller. Ultem gears displayed thickness values closest to the nominal dimension and the designed tolerance. The charts also suggest that following the completion of the load cycle, the thickness of PEEK gears was slightly changed in comparison to their condition after the printing process. This observation is confirmed by tooth topography charts, as tooth geometry was altered only slightly.

**Figure 10:**
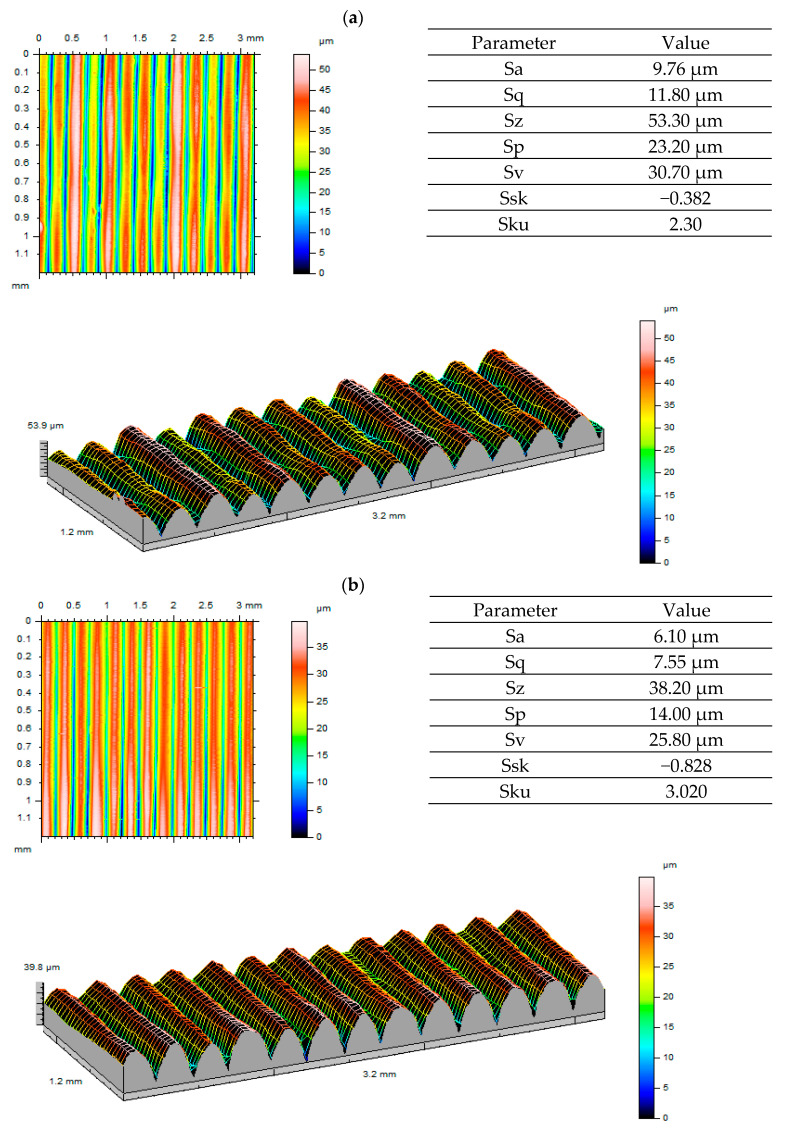
Roughness measurement results for the ABS material: (**a**) before testing; (**b**) after testing.

**Figure 11:**
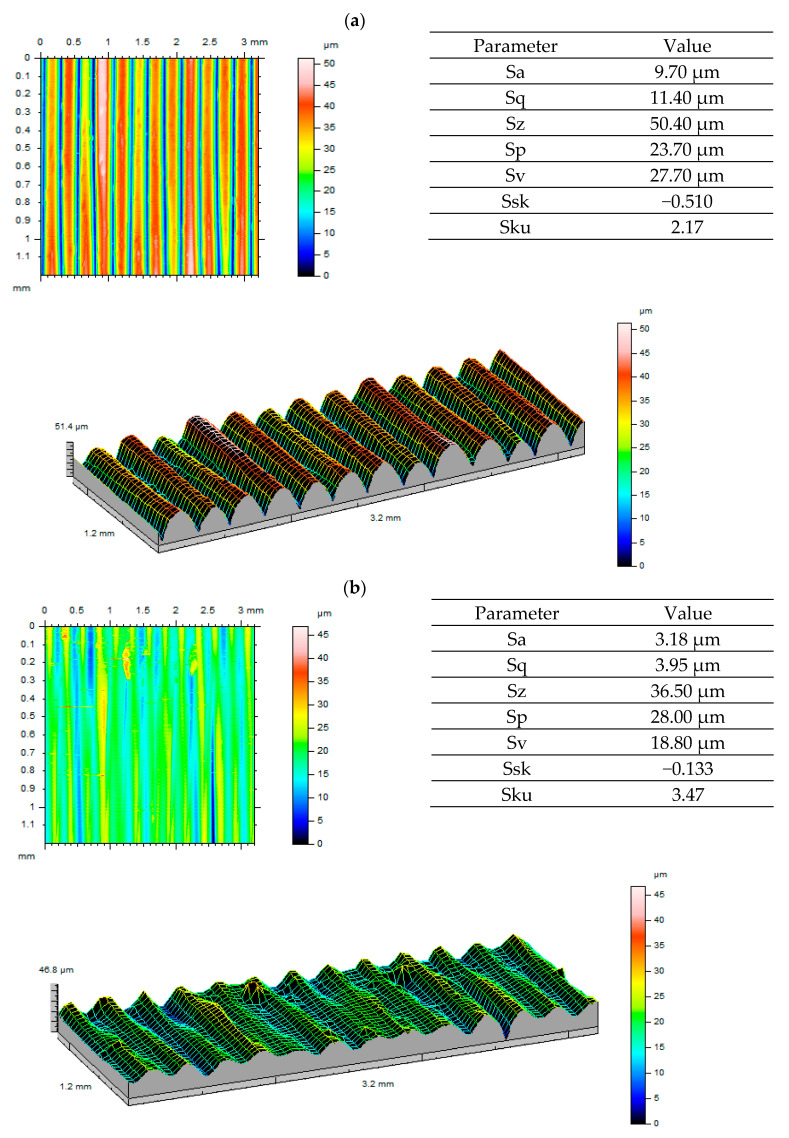
Roughness measurement results for the ULTEM material: (**a**) before testing; (**b**) after testing.

**Figure 12:**
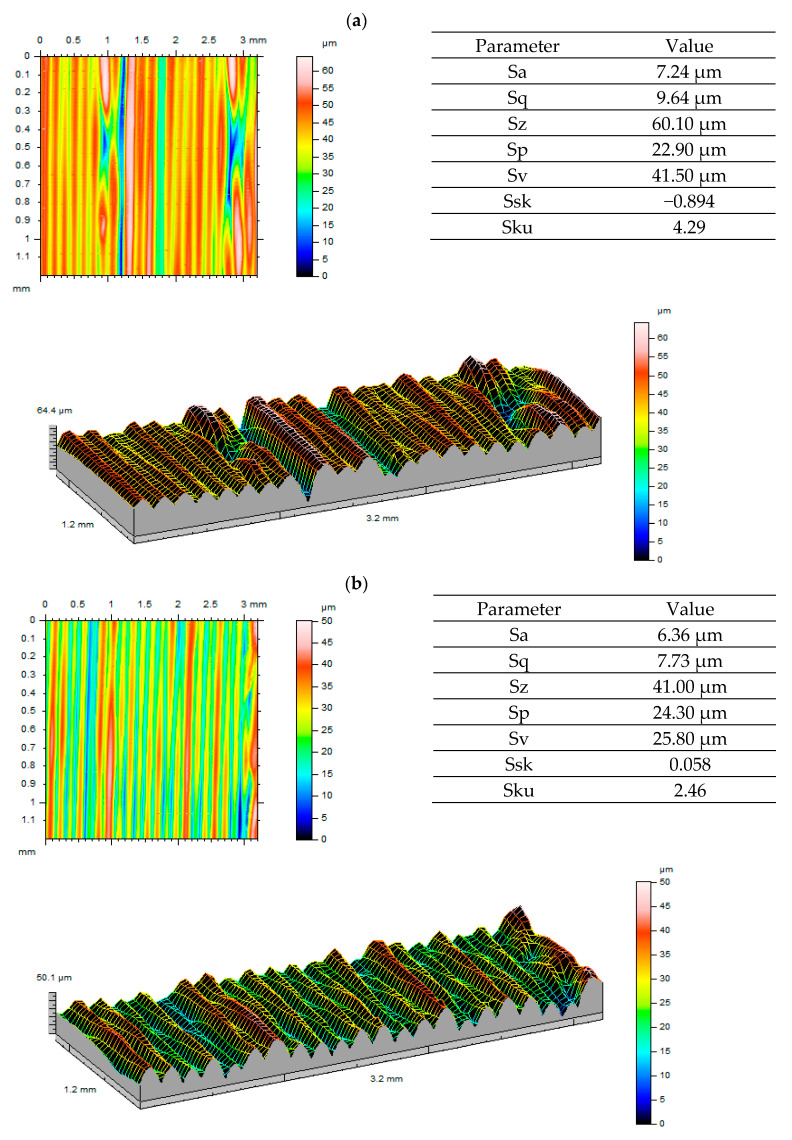
Roughness measurement results for the PEEK material: (**a**) before testing; (**b**) after testing.

Considering the visualisations generated and surface roughness values obtained before and after gear tests (Figure 10, Figure 11 and Figure 12), we can see that the gear surface wear process is most visible for ULTEM. This is confirmed both by 3D visualisations and reported parameters. With regard to Sa and Sq parameters, a decrease in values was observed. The most significant drop occurred for ULTEM. The value of parameter Sz, i.e., the sum of Sp and Sv, was also noticeably lower. This situation was clearly influenced by the tooth wear process as one gear meshed with another. Considering the asymmetry ratio, most analysed data are characterised by negative skewness. A platykurtic distribution can be observed when analysing the inclination ratio of output surfaces for ABS-M30 and ULTEM. The inclination value approaches normal distribution after tooth engagement tests. For the PEEK material, the output surface displayed leptokurtic distribution. This may have been caused by a non-uniform periodic structure forming after printing.

Gear hardness after the complete load cycle was also tested. The following instruments were used in the hardness tests:

- ZwickRoell H04.3106.01 (ISO 2039-1/2) [74,75] digital Rockwell hardness tester using loaded ball indentation.
- Shore D digital hardness tester (durometer), model HDD 100-1 (ISO 868) [76].

The measurements demonstrated that ABS M-30 and PEEK gears offer very similar properties: for pinions—3.54 and 3.42 N/mm^2^; for gears—3.51, 3.46 N/mm^2^, respectively. Due to excessive ball indentation in Ultem 9085 gears, hardness was measured by means of the Shore D technique. The value of hardness measured for both gears and pinions was 22.4. This is most likely caused by the absence of cross-linking in Ultem 9085 (the gears were not subjected to the annealing process) or changes which take place in the material due to the gear train’s working temperature. The maximum working temperature of gear trains read out for the last load cycle was around 60 °C for Ultem 9085, 55 °C for ABS M-30 and 30 °C for PEEK.

## 4. Conclusions

Tooth flank surface topography measurements enable tooth geometry assessment across the entire width of the toothed ring. This is of special importance in the evaluation of material distribution on the tooth flank in comparison to its required geometry expected at the design stage or during a key manufacturing phase (e.g., after hardening). It is another crucial aspect in polymer gear manufacturing, as it enables the assessment of the reproduction accuracy of the original model’s geometry, determining the correctness of the additive manufacturing or injection moulding in order to adjust the manufacturing process or model geometry—e.g., by allowing for shrinkage.

This study used topography charts to assess the level of wear on tooth flank surfaces of gears made from polymer materials made by additive FDM and FFF manufacturing techniques. It was reported that gears made of ABS M-30, Ultem 9085 and PEEK undergo wear to varying degrees. The active flank of PEEK gear teeth was worn to the smallest extent. This is confirmed by tooth topography charts as well as the image of the geometrical surface structure. The tooth thickness analysis indicated that the PEEK gear manufacturing process does not guarantee the stability of the gear’s geometry (thickness fluctuations outside tolerance, especially the lower deviation). The fluctuations of the thickness of teeth of ABS M-30 and Ultem 9085 gears are definitely (nearly twice as) lower. The topographies of pinions (z = 17) and gear wheels (z = 21) made from ABS M-30 and PEEK display similar changes, whereas the topography of Ultem 9085 pinions (z = 17) is entirely different. A convexity is present on the active tooth flank; it is observable across the width of the toothed ring. The topography of gear wheels (not presented in this study) made from Ultem 9085 is concave. It is presumably caused by the effect of the gear drive working temperature, which leads to altered physical properties (hardness) of the material.

Gear wear experiments and analysis clearly suggest that PEEK gears are the most resistant to wear. Surprising results were obtained for Ultem 9085, which was characterised by the greatest wear resulting from a change in its hardness. ABS M-30 gears were worn to a lesser extent than Ultem gears. Further analyses are necessary to determine the durability of gears made from materials subjected to thermal processing and modifications to the parent material.

## Figures and Tables

**Table 1:** Parameters of gears used in test rig.

Parameter	Driving Gear	Driven Gear
Number of teeth	17	21
Pressure angle (deg)	20.000	
Module (mm)	3.000	
Profile shift coefficient	0.000	0.000
Dedendum coefficient of the basic rack profile	1.250	1.250
Root radius coefficient of the basic rack profile	0.200	0.200
Addendum coefficient of the basic rack profile	1.000	1.000
Centre distance (mm)	57.000	
Face width (mm)	14.000	12.000
Outside diameter (mm)	57.000	69.000
Base tangent length (no backlash) (mm)	$22.855_{-0.282}^{+0.094}$	$23.023_{-0.282}^{+0.094}$
Normal backlash (mm)	0.188	

**Table 2:** Selected properties of materials used in 3D-printed gears [63,64,65,66].

Parameter/Material	ABS M-30	ULTEM 9085	PEEK (360–400)
Continuous operation temperature	up to 85 °C	up to 170 °C	up to 260 °C
Tensile Strength, Yield (ASTM D638) (ISO 527—PEEK)	31 MPa	77.1 MPa	105 MPa
Modulus (Elastic) (ASTM D638) (ISO 527—PEEK)	2.4 GPa	2.54 GPa	4.1 GPa
Flexural Strength (ASTM D790) (ISO 604—PEEK)	60 MPa	98.3 MPa	130 MPa
IZOD Impact, notched (ASTM D256) (ISO 180/A—PEEK)	101 J/m	73.7 J/m	5 kJ/m^2^
Shore D Hardness (ASTM D2240) (ISO 868—PEEK)	100	95–99	85–95
HDT—Heat Distortion Temperature at 1.82 MPa (ASTM D648) (ISO 75A-f—PEEK)	100 °C	172.9 °C	156 °C

**Table 3:** Information of prints made using additive manufacturing.

Additive Manufacturing	FDM	FDM	FFF
Printer	Stratasys F170	Fortus 450mc (Stratasys)	3DGence Industry F340
Material (brand name)	ABS M-30 ivory	ULTEM 9085 resin black	PEEK natural (360–400)
General type of material	ABS—Acrylonitrile Butadiene Styrene	PEI—Polyetherimide	PEEK—Polyetheretherketone
Processing temp.	250 °C–260 °C	ca. 380 °C	ca. 410 °C
Fill material	ca. 100%	100%	80%
Layer thickness	0.254 mm	0.254 mm	0.15 mm
Support material	QSR	ULTEM 9085 resin support	ESM-10 3DGence
Post-processing	Soluble	Breakaway	Soluble

**Table 4:** Parameters used to measure the gears.

Measuring Machine	Klingelnberg Gear Measuring Centre P40
Probe System	K3D (M44)
Resolution	<0.01 μm
Probe	D = 1.5 mm
Length measurement uncertainty	according to VDI/VDE 2617 U_1_ = 1.8 + L/250 [μm] L—length in mm
Teeth to be checked (profile, lead)	3 teeth (evenly around the gear circumference)
